# 3M™ Petrifilm™ Rapid Yeast and Mold Count Plate Method for the Enumeration of Yeast and Mold on Selected Surfaces: AOAC Official Method 2014.05

**DOI:** 10.1093/jaoacint/qsac118

**Published:** 2022-09-30

**Authors:** Cari K Lingle, April J Schumacher, Micki L Rosauer, Karen M Silbernagel, Jonathan Blackburn

**Affiliations:** 3M Company, Food Safety Department, St. Paul, MN 55144-1000, USA; 3M Company, Food Safety Department, St. Paul, MN 55144-1000, USA; 3M Company, Food Safety Department, St. Paul, MN 55144-1000, USA; 3M Company, Food Safety Department, St. Paul, MN 55144-1000, USA; Q Laboratories, Cincinnati, OH 45204, USA

## Abstract

**Background:**

The 3M™ Petrifilm™ Rapid Yeast and Mold Count (RYM) Plate contains nutrients supplemented with antibiotics, a cold-water-soluble gelling agent, and an indicator system that facilitates yeast and mold enumeration in 48–60 h.

**Objective:**

The objective of this study is to evaluate the 3M Petrifilm RYM Plate in a matrix extension study for the enumeration of yeast and mold on stainless steel, sealed concrete, and rubber surfaces.

**Methods:**

The 3M Petrifilm RYM Plate was compared to the U.S. Food and Drug Administration *Bacteriological Analytical Manual*, Ch. 18: *Yeasts, Molds and Mycotoxins* in a paired matrix study for enumeration of yeast and mold on stainless steel, sealed concrete, and rubber environmental surfaces.

**Results:**

The 3M Petrifilm RYM Plate demonstrated equivalent performance to the reference method for enumeration of yeast and mold from stainless steel, sealed concrete, and rubber environmental surfaces. There were no significant statistical differences between the 3M Petrifilm RYM Plate and reference method results for the three matrixes evaluated.

**Conclusion:**

The 3M Petrifilm RYM Plate is an effective plating method for enumerating yeast and mold when analyzing stainless steel, sealed concrete, and rubber surfaces.

**Highlights:**

The 3M Petrifilm RYM Plate method allows the user to obtain accurate results within 48–60 h in the matrices evaluated for the presence of yeast and mold when incubated at 25 ± 1°C or 28 ± 1°C. Interpretation and colony counting was straightforward and the 3M Petrifilm RYM Plate method required no additional agar or Petri dishes, creating an easier workflow by cutting down on supplies, sample plating time, and most noticeably, occupying less space in the incubator.

Foodborne yeasts and molds constitute a large and diverse group of microorganisms. Due to their heterotrophic nature, these microorganisms are able to adapt and survive in a wide range of environmental conditions including acidic and alkaline conditions, temperatures ranging from 5°C to 35°C, and low-moisture (<0.85_Aw_) products (1, 2). Many of these species cause varying degrees of food decomposition leading to substantial economic losses. Several foodborne molds, and possibly some yeasts, may also be hazardous to human health due to their ability to produce toxic metabolites (1). The presence of these microorganisms in food commodities can indicate contamination of the product, inadequately cleaned food processing equipment, or inadequate food storage facilities (2).

The 3M™ Petrifilm™ RYM Plate is a sample-ready culture medium system which contains nutrients supplemented with antibiotics, a cold-water-soluble gelling agent, and an indicator system that facilitates yeast and mold enumeration but does not differentiate species of yeast and mold from one another.

Yeasts appear as small, pink/tan or blue/green colonies, raised with defined edges and uniform in color. Molds appear as large, flat, blue/green colonies with a dark center and diffuse edges. The plates are incubated at 25 ± 1°C or 28 ± 1°C for 48 to 60 h.

The 3M Petrifilm RYM Plate was previously shown to be comparable to ISO 21527:2008 parts 1 and 2, and the U.S. Food and Drug Administration *Bacteriological Analytical Manual* (BAM) Ch. 18, Yeasts, Molds and Mycotoxins reference methods for the enumeration of yeasts and molds in foods (3–5), and AOAC SMPR 2021.009 for Viable Total Yeast and Mold Count Enumeration from dried cannabis flower (6) at 25 ± 1°C and 28 ± 1°C after 48 to 60 h for foods or 60 to 72 h for dried cannabis flower (7–10).

This matrix extension study compares the 3M Petrifilm RYM Plate method to the BAM Ch. 18 method for the enumeration of yeast and mold on stainless steel, sealed concrete, and rubber.

## Matrix Extension Validation Study

This study was conducted under the AOAC *Performance Tested Methods*^SM^ program and the *AOAC INTERNATIONAL Methods Committee Guidelines for Validation of Microbiological Methods for Food and Environmental Surfaces* (11) by Q Laboratories (Cincinnati, OH, USA) comparing the 3M Petrifilm RYM Plate method to the BAM Ch. 18 reference method using a paired study design for stainless steel (18 GA, 300 series, brushed finish), sealed concrete, and rubber (ethylene propylene diene monomer).

### Matrix Study

Stainless steel, rubber, and sealed concrete were provided by the independent laboratory and chemically disinfected with an Environmental Protection Agency-regulated quaternary ammonium solution. The solution was added to the surface and remained wet for 10 min for proper disinfection according to the product technical datasheet. After 10 min the excess liquid was removed, and the surface was allowed to dry. After disinfection, the environmental surfaces were artificially contaminated using organisms obtained from the American Type Culture Collection (ATCC^®^, Manassas, VA, USA) and Q Laboratories (QL) as follows: *Aspergillus flavus* ATCC 9643 (source: shoe sole) was used to inoculate stainless steel, *Penicillium chrysogenum* ATCC 10106 (source: cheese) was used to inoculate rubber, and *Candida lusitaniae* QL 15166–2 (source: beverage) was used to inoculate sealed concrete.

The study consisted of evaluating a total of 20 paired 100 cm^2^ samples of stainless steel, 20 paired 100 cm^2^ samples of rubber, and 20 paired 100 cm^2^ samples of sealed concrete. Within each sample set of 20, there were five uninoculated samples (0 cfu/100 cm^2^), five samples inoculated at 1–100 cfu/100 cm^2^, five samples inoculated at 100–1000 cfu/100 cm^2^, and 5 samples inoculated at 1000–10 000 cfu/100 cm^2^. All test portions were analyzed using the 3M Petrifilm RYM Plates and the BAM Ch 18 reference method.

The mold cultures for *Aspergillus flavus* and *Penicillium chrysogenum* were propagated in a vented tissue culture flask containing potato dextrose agar (PDA) from a frozen stock spore suspension stored at −70°C. The agars were incubated for 7 days at 25 ± 1°C. The mold spore suspensions were prepared by rinsing the tissue culture flask twice with 20 mL sterile saline (0.9%), with 0.05% Tween 80. The mold suspensions were then diluted to the appropriate levels for inoculation.

The initial yeast culture for *Candida lusitaniae* was propagated on Sabouraud dextrose agar (SDA) from a stock culture stored at −70°C. The SDA was incubated at 25 ± 1°C for 48–72 h before transferring a single colony to Sabouraud dextrose broth (SDB) broth and incubating at 25 ± 1°C for 48–72 h. The stock culture was diluted to a volume that allowed for even distribution of inoculum over entire 100 cm^2^ test surface area, but without producing excessive accumulation of liquid that dried unevenly (0.25 mL/100 cm^2^).

After inoculation, the surface was allowed to dry for 16–24 h at room temperature (18–25°C). The surface was visually dry at the time of sampling. All test areas were sampled using horizontal and vertical strokes using firm pressure while rotating the head of the swab to ensure the entire area was sampled. For this study, the swabs were placed back into their original containers and held at room temperature (18–25°C) for 2 h. Swabs were thoroughly homogenized by vortex mixing prior to performing dilutions and plating.

### Candidate Method

For the 3M Petrifilm RYM Plate candidate method, the paired 100 cm^2^ test portions of stainless steel, sealed concrete, and rubber were sampled using the 3M Swab Sampler with 10 mL Letheen broth. All test areas were sampled using horizontal and vertical strokes with firm pressure while rotating the head of the swab to ensure the entire area was sampled. The 3M Swab Samplers were put back into their original containers and thoroughly homogenized by vortex mixing. For this study, swab samples were held at room temperature (18–25°C) for 2 h before plating. After 2 h, each sample was homogenized by vortex mixing for 1 min.

A 1:10 serial dilution was conducted by transferring 1 mL product into 9 mL Butterfield’s phosphate buffer (BPBD). The appropriate serial dilutions were conducted from each swab sample in order to achieve counts within the countable range. A 1 mL aliquot of each dilution was plated onto the center of six separate 3M Petrifilm RYM Plates. The top film was lifted and with the pipette perpendicular, 1 mL sample suspension was dispensed onto the center of the bottom film. The top film was rolled down onto the sample. The 3M Petrifilm Flat Spreader was placed on the center of the plate and was gently pressed on the center of the spreader to distribute the sample evenly. The inoculum was spread over the entire 3M Petrifilm RYM Plate growth area before the gel formed. Care was taken to make sure the spreader did not slide across the film. The plates were left undisturbed for at least 1 min to permit the gel to form.

For each dilution, three of the plates were incubated at 25 ± 1°C and three of the plates were incubated at 28 ± 1°C in a horizontal position in stacks no greater than 40 plates. Plates were removed from incubation after 48 ± 2 h and typical yeast and mold colonies in the countable range (15–150) were enumerated using a standard colony counter. Plates containing greater than 150 colonies were either estimated or recorded as to numerous to count (TNTC). All plates were re-incubated at their respective temperatures for an additional 12 h (to reach a total incubation time of 60 h) and colonies were enumerated a second time in the same manner as for the 48 h time point.

### Reference Method

For the BAM Ch. 18 reference method, the dilutions from each surface preparation for the 3M Petrifilm RYM Plate method were also used for the reference method. From the initial dilution (1:10), 1 mL was distributed across three dichloran rose-bengal chloramphenicol (DRBC) agar plates in triplicate (3 mL across nine total plates). From the same dilution (1:10), 0.1 mL was plated onto three DRBC agar plates. From the 1:100 dilution tube, 0.1 mL was plated onto three DRBC agar plates. Inoculum was spread with a sterile, bent glass rod. Plates were incubated upright at 25 ± 1°C for 5 days.

After incubation, the DRBC plates were enumerated at the dilutions containing 10–150 cfu/plate. Plate count results were expressed in the average cfu per 100 cm^2^ based on the average count of the triplicate set, calculated to include the dilution factor of the plated dilution with colonies in the countable range.

**AOAC *Official Method*^SM^ 2014.05**

**Enumeration of Yeast and Mold in Foods, Selected Surfaces and DriedCannabis Flower**

**3M™ Petrifilm™ Rapid Yeast and Mold Count Plate**

**First Action 2014**

**Final Action 2017**

**Revised First Action 2022 (for Cannabis Flower, THC > 0.3%, Only)**

[Applicable to the enumeration of yeast and mold in the following high-water activity matrixes: yogurt, frozen bread dough, fermented salami, sour cream, ready-made pie, raw frozen ground beef patties (77% lean), ready-to-eat deli sandwiches, sliced apples, and the following low-water activity matrixes: raw almonds, dehydrated soup, dried cannabis flower (THC > 0.3%), and the following environmental surfaces: stainless steel, sealed concrete, and rubber.]

*Caution*: After use, the diluents and 3M Petrifilm RYM Plates may contain microorganisms that may be a potential biohazard as several foodborne molds have the ability to produce toxic metabolites known as mycotoxins. If further identification of a mold species is required, appropriate personal protective equipment (PPE) should be used when top film is retracted and exposure to spores or mycotoxins may occur. When testing is complete, follow current industry standards for the disposal of contaminated waste. Consult the material safety data sheet for additional information and local regulations for disposal. For information on potential biohazards, reference *Biosafety in Microbiological and Biomedical Laboratories*, 6th Ed., Section VIII-B: Fungal Agents.

The 3M Petrifilm RYM Plates contain chloramphenicol and chlortetracycline, potent broad spectrum antibiotic drugs commonly used in yeast and mold enumeration. The drugs, when used in humans, are associated with many toxic effects. Care should be taken to avoid coming into direct contact with the gel on the plates.

*See* Tables **2014.05A** and 2014.05B for a summary of results of the collaborative study. The result for each collaborating laboratory’s aerobic plate count analysis for each matrix is shown in Table **2014.05C**.

*See* Tables 2–9 for detailed results of the collaborative study [*J. AOAC Int.* **98**, 767 (2015)].

**Table 1.** Matrix study: 3M Petrifilm RYM Plate (25°C 48 h) versus FDA BAM Ch. 18 results

Matrix	Contamination level^c^	3M Petrifilm RYM Plate results		FDA BAM Ch. 18 results^a^		DOM^f^	SE^g^	90% CI^b^		95% CI	
		Mean^d^	s_r_^e^	Mean	s_r_			LCL^h^	UCL^i^	LCL	UCL
Stainless steel/*Aspergillus flavus* ATCC^j^ 9643	Non	0	0	0	0	0	0	0	0	0	0
	Low	1.004	0.000	1.124	0.164	−0.120	0.073	−0.276	0.037	−0.323	0.084
	Med	2.054	0.061	2.445	0.055	−0.391	0.037	−0.461	−0.321	−0.478	−0.304
	High	3.044	0.076	3.254	0.105	−0.211	0.031	−0.278	−0.144	−0.298	−0.123
Rubber/*Penicillium chrysogenum* ATCC 10106	Non	0	0	0	0	0	0	0	0	0	0
	Low	1.745	0.066	1.745	0.066	0.000	0.021	−0.045	0.045	−0.059	0.059
	Med	2.794	0.109	2.811	0.115	−0.018	0.054	−0.132	0.096	−0.166	0.131
	High	3.320	0.088	3.440	0.173	−0.121	0.057	−0.242	0.001	−0.279	0.038
Sealed concrete/*Candida lusitaniae* QL^k^ 15166–2	Non	0	0	0	0	0	0	0	0	0	0
	Low	1.752	0.256	1.722	0.250	0.029	0.145	−0.281	0.339	−0.374	0.433
	Med	2.691	0.182	2.623	0.190	0.068	0.058	−0.056	0.193	−0.094	0.230
	High	3.748	0.146	3.727	0.143	0.021	0.063	−0.114	0.156	−0.155	0.197

a FDA BAM Ch. 18, Yeasts, Molds and Mycotoxins.

b CI = Confidence interval.

c All surfaces are artificially contaminated, 100 cm^2^ test areas. Non indicates non-inoculated.

d Mean of five replicate portions, after logarithmic transformation: Log_10_[cfu/g + (0.1)f] where f is the smallest reportable result.

e Repeatability standard deviation.

f DOM = Difference of means between the candidate and reference methods.

g SE = Standard error.

h Lower confidence limit for difference of means.

i Upper confidence limit for difference of means.

J American Type Culture Collection, Manassas, VA. USA.

k Q Laboratories Culture Collection, Cincinnati, OH, USA.

**Table 2.** Matrix study: 3M Petrifilm RYM Plate (25°C 60 h) versus FDA BAM Ch. 18 results

Matrix	Contamination level^c^	3M Petrifilm RYM Plate results		FDA BAM Ch. 18 results^a^		DOM^f^	SE^g^	90% CI^b^		95% CI	
		Mean^d^	s_r_^e^	Mean	s_r_			LCL^h^	UCL^i^	LCL	UCL
Stainless steel/*Aspergillus flavus* ATCC^j^ 9643	Non	0	0	0	0	0	0	0	0	0	0
	Low	1.004	0.000	1.124	0.164	−0.120	0.073	−0.276	0.037	−0.323	0.084
	Med	2.086	0.030	2.445	0.055	−0.359	0.027	−0.416	−0.301	−0.434	−0.284
	High	3.134	0.078	3.254	0.105	−0.120	0.039	−0.203	−0.037	−0.228	−0.012
Rubber/*Penicillium chrysogenum* ATCC 10106	Non	0	0	0	0	0	0	0	0	0	0
	Low	1.772	0.083	1.745	0.066	0.027	0.034	−0.044	0.099	−0.066	0.121
	Med	2.786	0.093	2.811	0.115	−0.026	0.056	−0.146	0.094	−0.182	0.130
	High	3.405	0.061	3.440	0.173	−0.035	0.056	−0.155	0.085	−0.191	0.121
Sealed concrete/*Candida lusitaniae* QL^k^ 15166–2	Non	0	0	0	0	0	0	0	0	0	0
	Low	1.761	0.265	1.722	0.250	0.038	0.149	−0.280	0.356	−0.376	0.452
	Med	2.690	0.179	2.623	0.190	0.068	0.059	−0.058	0.193	−0.096	0.231
	High	3.742	0.140	3.727	0.143	0.015	0.059	−0.111	0.141	−0.150	0.179

a FDA BAM Ch. 18, Yeasts, Molds and Mycotoxins.

b CI = Confidence interval.

c All surfaces are artificially contaminated, 100 cm^2^ test areas. Non indicates non-inoculated.

d Mean of five replicate portions, after logarithmic transformation: Log_10_[cfu/g + (0.1)f] where f is the smallest reportable result.

e Repeatability standard deviation.

f DOM = Difference of means between the candidate and reference methods.

g SE = Standard error.

h Lower confidence limit for difference of means.

i Upper confidence limit for difference of means.

J American Type Culture Collection, Manassas, VA, USA.

k Q Laboratories Culture Collection, Cincinnati, OH, USA.

**Table 3.** Matrix study: 3M Petrifilm RYM Plate (28°C 48 h) versus FDA BAM Ch. 18 results

Matrix	Contamination level^c^	3M Petrifilm RYM Plate results		FDA BAM Ch. 18 results^a^		DOM^f^	SE^g^	90% CI^b^		95% CI	
		Mean^d^	s_r_^e^	Mean	s_r_			LCL^h^	UCL^i^	LCL	UCL
Stainless steel/*Aspergillus flavus* ATCC^j^ 9643	Non	0	0	0	0	0	0	0	0	0	0
	Low	1.004	0.000	1.124	0.164	−0.120	0.073	−0.276	0.037	−0.323	0.084
	Med	2.105	0.050	2.445	0.055	−0.339	0.034	−0.411	−0.268	−0.433	−0.246
	High	3.109	0.111	3.254	0.105	−0.145	0.037	−0.225	−0.066	−0.249	−0.042
Rubber/*Penicillium chrysogenum* ATCC 10106	Non	0	0	0	0	0	0	0	0	0	0
	Low	1.761	0.062	1.745	0.066	0.016	0.035	−0.059	0.090	−0.081	0.113
	Med	2.781	0.100	2.811	0.115	−0.030	0.047	−0.129	0.069	−0.159	0.099
	High	3.312	0.101	3.440	0.173	−0.128	0.070	−0.278	0.022	−0.323	0.067
Sealed concrete/*Candida lusitaniae* QL^k^ 15166–2	Non	0	0	0	0	0	0	0	0	0	0
	Low	1.788	0.232	1.722	0.250	0.066	0.055	−0.053	0.184	−0.088	0.220
	Med	2.723	0.144	2.623	0.190	0.100	0.055	−0.017	0.217	−0.053	0.252
	High	3.627	0.301	3.727	0.143	−0.100	0.097	−0.306	0.106	−0.368	0.168

a FDA BAM Ch. 18, Yeasts, Molds and Mycotoxins.

b CI = Confidence interval.

c All surfaces are artificially contaminated, 100 cm^2^ test areas. Non indicates non-inoculated.

d Mean of five replicate portions, after logarithmic transformation: Log_10_[cfu/g + (0.1)f] where f is the smallest reportable result.

e Repeatability standard deviation.

f DOM = Difference of means between the candidate and reference methods.

g SE = Standard error.

h Lower confidence limit for difference of means.

i Upper confidence limit for difference of means.

j American Type Culture Collection, Manassas, VA, USA.

k Q Laboratories Culture Collection, Cincinnati, OH, USA.

**Table 4.** Matrix study: 3M Petrifilm RYM Plate (28°C 60 h) vs FDA BAM Ch 18 results

Matrix	Contamination level^c^	3M Petrifilm RYM Plate results		FDA BAM Ch 18 results^a^		DOM^f^	SE^g^	90% CI^b^		95% CI	
		Mean^d^	s_r_^e^	Mean	s_r_			LCL^h^	UCL^i^	LCL	UCL
Stainless steel/*Aspergillus flavus* ATCC^j^ 9643	Non	0	0	0	0	0	0	0	0	0	0
	Low	1.004	0.000	1.124	0.164	−0.120	0.073	−0.276	0.037	−0.323	0.084
	Med	2.164	0.085	2.445	0.055	−0.281	0.042	−0.370	−0.192	−0.397	−0.165
	High	3.154	0.103	3.254	0.105	−0.100	0.030	−0.164	−0.036	−0.183	−0.017
Rubber/*Penicillium chrysogenum* ATCC 10106	Non	0	0	0	0	0	0	0	0	0	0
	Low	1.766	0.119	1.745	0.066	0.021	0.049	−0.083	0.126	−0.115	0.158
	Med	2.772	0.105	2.811	0.115	−0.040	0.045	−0.136	0.057	−0.166	0.086
	High	3.341	0.119	3.440	0.173	−0.099	0.074	−0.256	0.058	−0.303	0.105
Sealed concrete/*Candida lusitaniae* QL^k^ 15166–2	Non	0	0	0	0	0	0	0	0	0	0
	Low	1.788	0.232	1.722	0.250	0.066	0.055	−0.053	0.184	−0.088	0.220
	Med	2.735	0.149	2.623	0.190	0.112	0.058	−0.011	0.236	−0.049	0.273
	High	3.723	0.168	3.727	0.143	−0.005	0.039	−0.087	0.078	−0.111	0.102

a FDA BAM Ch 18, Yeasts, Molds and Mycotoxins.

b CI = Confidence interval.

c All surfaces are artificially contaminated, 100 cm^2^ test areas. Non indicates non-inoculated.

d Mean of five replicate portions, after logarithmic transformation: Log_10_[cfu/g + (0.1)f] where f is the smallest reportable result.

e Repeatability standard deviation.

f DOM = Difference of means between the candidate and reference methods.

g SE = Standard error.

h Lower confidence limit for difference of means.

i Upper confidence limit for difference of means.

j American Type Culture Collection, Manassas, VA, USA.

k Q Laboratories Culture Collection, Cincinnati, OH, USA.

**Table 2014.05A.** Interlaboratory study results of 3M Petrifilm RYM versus FDA BAM and ISO 21527 methods for frozen raw ground beef patties

		3M Petrifilm RYM method				BAM/ISO 21527 methods^a^					Difference of means	Reverse transformed mean difference^e^
Matrix	Lot	*n* ^b^	Mean^c^	s_r_	s_R_	*n*	Mean	s_r_	s_R_	*P*-value^d^		
Frozen raw ground beef patties												
25°C, 48 h	Control	11(0)	<1.00	—^f^	—	11(0)	<1.00	—	—	—	—	—
	Low	11(0)	2.12	0.41	0.41	11(1)	2.07	0.36	0.38	0.5323	0.05	14.34
	Medium	11(0)	3.52	0.10	0.10	11(0)	3.47	0.09	0.11	0.1637	0.05	360.10
	High	11(0)	4.65	0.13	0.14	11(0)	4.59	0.10	0.14	0.2266	0.06	5763.84
25°C, 60 h	Control	11(0)	<1.00	—	—	11(0)	<1.00	—	—	—	—	—
	Low	11(0)	2.14	0.36*^g^*	0.37	11(1)	2.07	0.36	0.38	0.3773	0.07	20.55
	Medium	11(0)	3.52	0.10	0.10	11(0)	3.47	0.09	0.11	0.1573	0.05	360.10
	High	11(0)	4.65	0.14	0.15	11(0)	4.59	0.10	0.14	0.1750	0.06	5763.84
28°C, 48 h	Control	11(0)	<1.00	—	—	11(0)	<1.00	—	—	—	—	—
	Low	11(0)	2.17	0.29*^g^*	0.30	11(1)	2.07	0.36	0.38	0.1391	0.10	30.42
	Medium	11(0)	3.53	0.10	0.10	11(0)	3.47	0.09	0.11	0.0824	0.06	437.23
	High	11(0)	4.67	0.08*^g^*	0.11	11(0)	4.59	0.10	0.14	0.0966	0.08	7869.00
28°C, 60 h	Control	11(0)	<1.00	—	—	11(0)	<1.00	—	—	—	—	—
	Low	11(0)	2.16	0.29*^g^*	0.29	11(1)	2.07	0.36	0.38	0.1843	0.09	27.05
	Medium	11(0)	3.53	0.09	0.10	11(0)	3.47	0.09	0.11	0.1095	0.06	437.23
	High	11(0)	4.67	0.08*^g^*	0.11	11(0)	4.59	0.10	0.14	0.1088	0.08	7869.00

^a^ Samples were analyzed by harmonized FDA-BAM Ch. 18 and ISO 21527 methods using 0.1% peptone water as the sample diluent.

b *n* = Number of laboratories that reported complete results. Outliers are in parentheses.

c Log_10_ yeast and mold cfu/g.

d Significant difference (*P* < 0.05).

e Results presented as cfu/g.

^f^ Not applicable.

g Results indicate that the candidate method is more repeatable than the reference methods. s_r_ = Repeatability standard deviation; s^R^ = reproducibility standard deviation.

**Table 2014.05B.** Interlaboratory study results of 3M Petrifilm RYM versus FDA BAM and ISO 21527 methods for raw almonds

		3M Petrifilm RYM method				FDA BAM/ISO 21527 methods^a^					Difference of means	Reverse transformed mean difference^*e*^
Matrix	Lot	*n^b^*	Mean^*c*^	s_r_	s_R_	*n*	Mean	s_r_	s_R_	*P*-value*^d^*		
Raw almonds												
25°C, 48 h	Control	12(0)	<1.00	—^f^	—	12(0)	<1.00	—	—	—	—	—
	Low	14(0)	1.45	0.17*^g^*	0.26	14(0)	1.55	0.19	0.34	0.4165	0.10	–7.30
	Medium	14(1)	2.12	0.26	0.39	14(0)	2.21	0.20	0.24	0.3322	0.09	–30.36
	High	14(2)	3.00	0.18	0.49	14(1)	3.08	0.12	0.31	0.2833	0.08	–202.26
25°C, 60 h	Control	12(0)	<1.00	—	—	12(0)	<1.00	—	—	—	—	—
	Low	14(0)	1.53	0.23	0.28	14(0)	1.55	0.19	0.34	0.8391	0.02	–1.60
	Medium	14(0)	2.20	0.21	0.27	14(0)	2.21	0.20	0.24	0.7789	0.01	–3.69
	High	14(2)	3.04	0.18	0.41	14(1)	3.08	0.12	0.31	0.5418	0.04	–105.79
28°C, 48 h	Control	12(0)	<1.00	—	—	12(0)	<1.00	—	—	—	—	—
	Low	14(0)	1.58	0.16*^g^*	0.21	14(0)	1.55	0.19	0.34	0.7381	0.03	2.54
	Medium	14(0)	2.17	0.17*^g^*	0.29	14(0)	2.21	0.20	0.24	0.6139	0.04	–11.73
	High	14(2)	3.01	0.17	0.45	14(1)	3.08	0.12	0.31	0.3904	0.07	–178.97
28°C, 60 h	Control	12(0)	<1.00	—	—	12(0)	<1.00	—	—	—	—	—
	Low	14(0)	1.60	0.17*^g^*	0.20	14(0)	1.55	0.19	0.34	0.5474	0.05	4.33
	Medium	14(0)	2.21	0.17*^g^*	0.23	14(0)	2.21	0.20	0.24	0.9483	0.00	0.00
	High	14(2)	3.03	0.18	0.42	14(1)	3.08	0.12	0.31	0.4687	0.05	–130.75

^a^ Samples were analyzed by harmonized FDA BAM Ch. 18 and ISO 21527 methods using 0.1% peptone water as the sample diluent.

b *n* = Number of laboratories that reported complete results. Outliers are in parentheses.

c Log_10_ yeast and mold cfu/g.

d Significant difference (*P* < 0.05).

e Results presented as cfu/g.

f Not applicable.

g Results indicate that the candidate method is more repeatable than the reference methods. s_r_ = Repeatability standard deviation; s_R_ = reproducibility standard deviation.

**Table 2014.05C.** Results of aerobic plate count for collaborating laboratories

Lab	Frozen raw ground beef, cfu/g	Raw almonds, cfu/g
1	3.8 × 10^2^	6.0 × 10^1^
2	1.1 × 10^3^	6.0 × 10^2^
3	<10	3.0 × 10^1^
4	Not reported	Not reported
5	2.8 × 10^3^	2.8 × 10^1^
6	8.0 × 10^1^	2.2 × 10^1^
7	9.1 × 10^2^	1.6 × 10^2^
8	Not reported	Not reported
9	9.0 × 10^2^	2.0 × 10^2^
10	1.3 x 10^3^	4.0 × 10^2^
11	>2500	1.0 × 10^1^
12	Not reported	7.0 × 10^1^
13	9.5 × 10^1^	1.0 × 10^1^
14	7.3 × 10^2^	2.3 × 10^2^
15	3.7 × 10^2^	8.0 × 10^1^

**Table 2014.05D.** Appearance of yeast and mold on 3M Petrifilm RYM Plates

Yeast	Mold
Small colonies	Large colonies
Colonies have defined edges	Colonies have diffused edges
Pink/tan or blue/green in color	Blue/green to variable upon prolonged incubation
Colonies appear raised (3-dimensional)	Colonies appear flat
Colonies have a uniform color	Colonies have a dark center with diffused edges

### A. Principle

The 3M Petrifilm RYM Plate is a sample-ready culture medium system, which contains nutrients supplemented with antibiotics, a cold-water-soluble gelling agent, and an indicator system that facilitates yeast and mold enumeration. 3M Petrifilm RYM Plates are used for the enumeration of yeast and mold in as little as 48 h in the food and beverage industries and 60 to 72 h in the cannabis industry. 3M Food Safety is certified to ISO (International Organization for Standardization) 9001 for design and manufacturing.

### B. Apparatus and Reagents

- *3M Petrifilm Rapid Yeast and Mold Count Plate.*—Available from 3M Food Safety, St. Paul, MN, USA—Cat. No. 6475/6477.
- *Sterile diluents.—*0.1% peptone water (PW) or Butterfield’s phosphate buffer (BPBD).
- *Pipets.—*Capable of 1000 µL or a serological pipet.
- *Sterile pipet tips.—*Capable of 1000 µL.
- *Stomacher.—*Seward or equivalent.
- *Filter stomacher bags.*—Seward or equivalent.
- *3M Petrifilm Flat Spreader.—* Cat. No. 6425.
- *3M Swab Sampler with 10 mL Letheen broth.—*Cat. No. RS96010LET or equivalent.
- *Incubators.—*Capable of maintaining 25 ± 1°C and 28 ± 1°C and having a solid front to maintain a dark interior.
- *Refrigerator.—*Capable of maintaining 2–8°C, for storing the 3M Petrifilm RYM Plates.
- *Standard colony counter or illuminated magnifier.*

### C. General Instructions

- Store unopened 3M Petrifilm RYM Plate pouches refrigerated or frozen (–20 to 8°C/–4 to 46°F). Just prior to use, allow unopened pouches to come to room temperature before opening (20–25°C/<60% RH). Return unused 3M Petrifilm RYM Plates to the pouch. Seal by folding the end of the pouch over and applying adhesive tape. To prevent exposure to moisture, do not refrigerate opened pouches. Store resealed pouches in a cool dry place (20–25°C/<60% RH) for no longer than 4 weeks. It is recommended that resealed pouches of 3M Petrifilm RYM Plates be stored in a freezer if the laboratory temperature exceeds 25°C (77°F) and/or the laboratory is located in a region where the relative humidity (RH) exceeds 60% (with the exception of air-conditioned premises).
  To store opened pouches in a freezer, place 3M Petrifilm RYM Plates in a sealable container.
  Post-incubation 3M Petrifilm RYM Plates can be stored at –10 to –20°C for up to 7 days.
- Follow all instructions carefully. Failure to do so may lead to inaccurate results.

### D. Sample Preparation

- Aseptically prepare a 1:10 dilution of each test portion.
  1. *Dairy products*.—Pipet 11 mL or weigh 11 g sample into 99 mL sterile 0.1% PW. Shake 25 times to homogenize.
  2. *All other foods.—*Weigh out 25 g sample from test portion into a sterile stomacher bag and dilute with 225 mL 0.1% PW; stomach at high speed to homogenize.
  3. *Dried cannabis flower (THC > 0.3*%*).—*Weigh out 10 g sample from test portion into a sterile stomacher bag and dilute with 90 mL sterile 0.1% PW. Shake 25 times to homogenize.
  4. *Environmental surfaces*.*—*Mix or shake swab in Letheen broth vigorously.
- Prepare 10-fold serial dilutions in 0.1% PW or BPBD. Environmental surface samples may be plated directly as needed.
- Place a 3M Petrifilm RYM Plate on a flat, level surface for each dilution to be tested.
- Lift the top of the film. Dispense 1 mL of each dilution onto the center of the bottom film of each plate.
- Roll the film down onto the sample.
- Place the 3M Petrifilm Flat Spreader on the center of the plate. Press gently on the center of the spreader to distribute the sample evenly. Spread the inoculum over the entire 3M Petrifilm RYM Plate growth area before the gel is formed. *Do not slide the spreader across the film.*
- Remove the spreader and leave the plate undisturbed for at least 1 min to permit the gel to form.
- Incubate the 3M Petrifilm RYM Plates at 25 or 28°C in a horizontal position with the clear side up in stacks of no more than 40.
  1. *For food or environmental samples*.*—*Enumerate plates after 48 h of incubation. If colonies appear faint, allow for an additional 12 h of incubation time for enhanced interpretation. If a 60 h time point for interpretation is not convenient, extending the incubation time to 72 h is an acceptable alternative.
  2. *For dried cannabis flower*.*—*Enumerate plates at 60 to 72 h of incubation.
- 3M Petrifilm RYM Plates can be counted using a standard colony counter with the use of a backlight or an illuminated magnifier to assist with the estimated enumeration. Do not count colonies on the foam dam since they are removed from the nutrient medium.
- Yeast colonies appear raised and small with defined edges. Colonies may appear pink/tan or blue/green in color.
- Mold colonies appear flat with a dark center and diffused edges. Colonies may appear blue/green to variable upon prolonged incubation. *See*Table **2014.05D** for yeast and mold appearance.
- The circular growth area is approximately 30 cm^2^. Plates containing greater than 150 colonies can be either estimated or recorded as TNTC. Estimation can only be done by counting the number of colonies in one or more representative squares and determining the average number per square. The average number can be multiplied by 30 to determine the estimated count per plate. If a more accurate count is required, the sample will need to be retested at higher dilutions. When the sample contains substantial amounts of mold, depending on the type of mold, the upper countable limit may be at user discretion.
- Samples may occasionally show interference on the 3M Petrifilm RYM Plates, for example:
  1. Uniform blue background color (often seen from the organisms used in cultured products). These should not be counted as TNTC.
  2. Intense pinpoint blue specks (often seen with spices, granulated products, or dried cannabis flower).
- Report final results as colony-forming units/gram (cfu/g).
- If required, colonies may be isolated for further identification by direct microscopy or biochemical analysis. Lift the top film and pick the colony from the gel.

### Results

**Figure 1.**
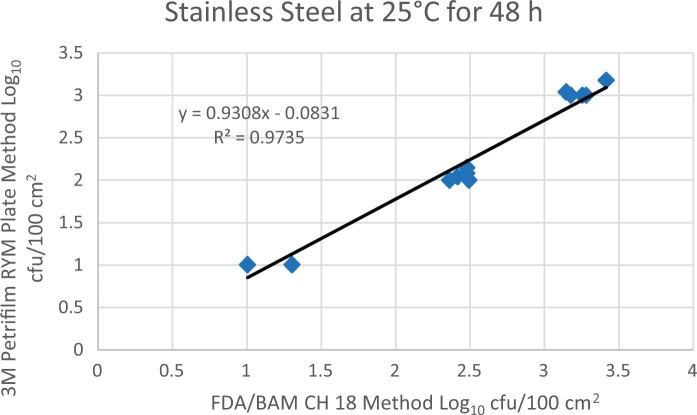
Method comparison results of 3M Petrifilm RYM Plate versus FDA BAM Ch. 18 for stainless steel at 25°C for 48 h.

**Figure 2.**
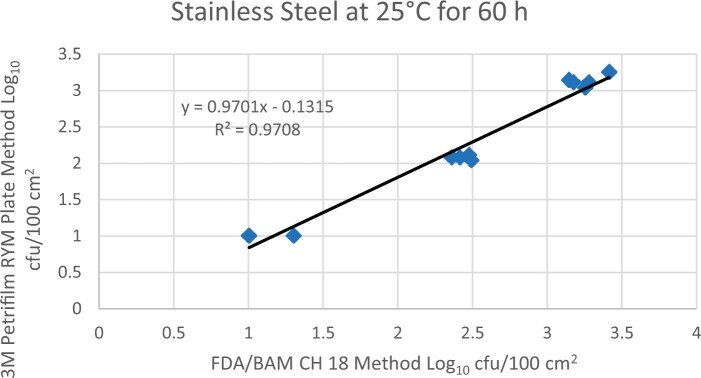
Method comparison results of 3M Petrifilm RYM Plate versus FDA BAM Ch. 18 for stainless steel at 25°C for 60 h.

**Figure 3.**
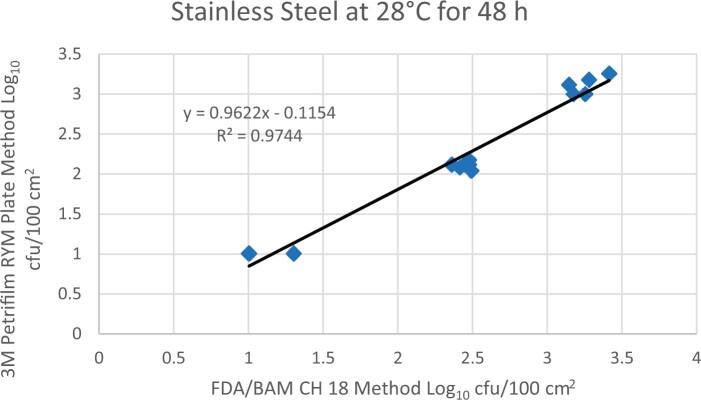
Method comparison results of 3M Petrifilm RYM Plate versus FDA BAM Ch. 18 for stainless steel at 28°C for 48 h.

**Figure 4.**
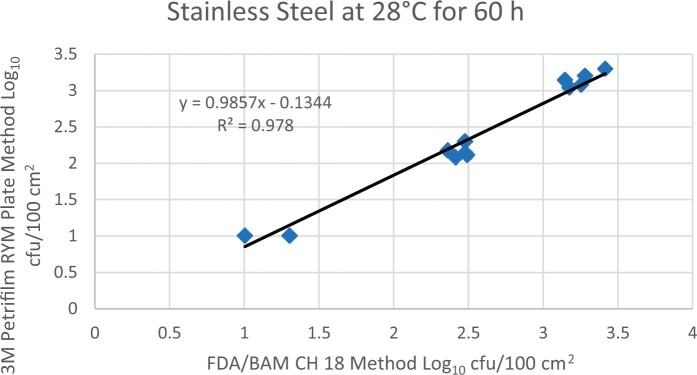
Method comparison results of 3M Petrifilm RYM Plate versus FDA BAM Ch. 18 for stainless steel at 28°C for 60 h.

**Figure 5.**
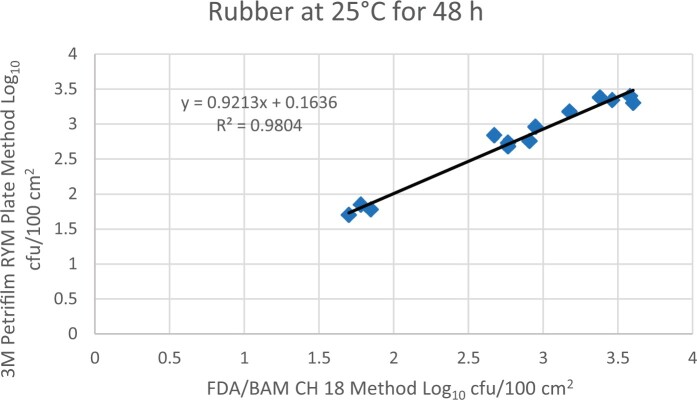
Method comparison results of 3M Petrifilm RYM Plate versus FDA BAM Ch. 18 for rubber at 25°C for 48 h.

**Figure 6.**
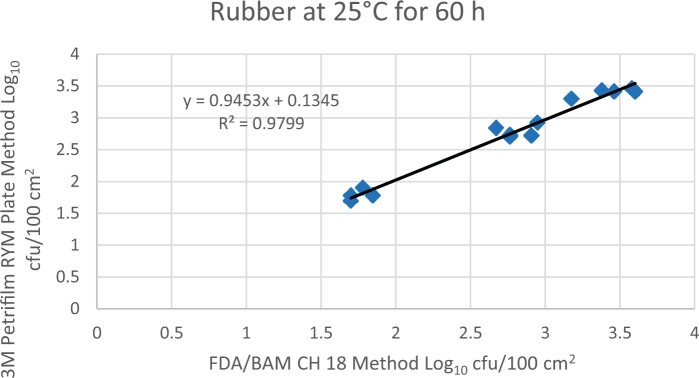
Method comparison results of 3M Petrifilm RYM Plate versus FDA BAM Ch. 18 for rubber at 25°C for 60 h.

**Figure 7.**
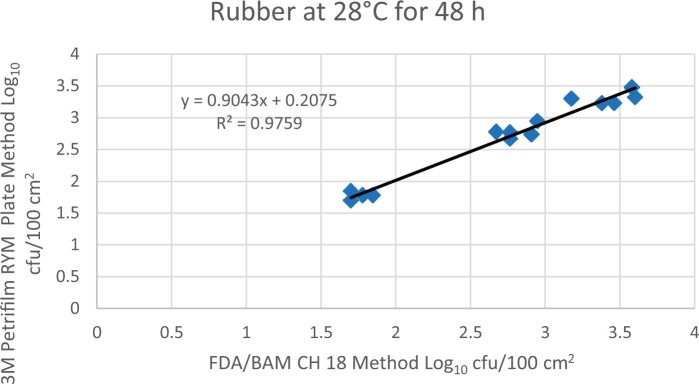
Method comparison results of 3M Petrifilm RYM Plate versus FDA BAM Ch. 18 for rubber at 28°C for 48 h.

**Figure 8.**
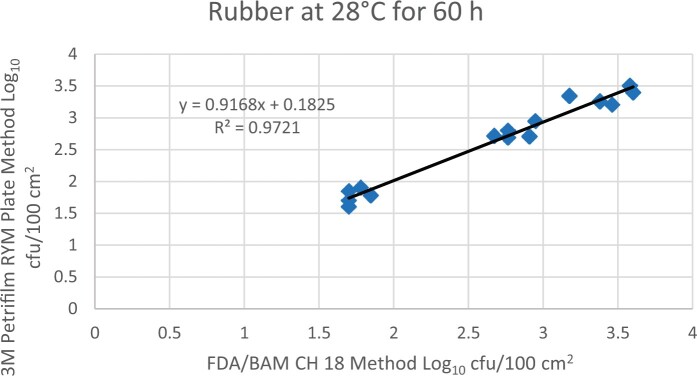
Method comparison results of 3M Petrifilm RYM Plate versus FDA BAM Ch. 18 for rubber at 28°C for 60 h.

**Figure 9.**
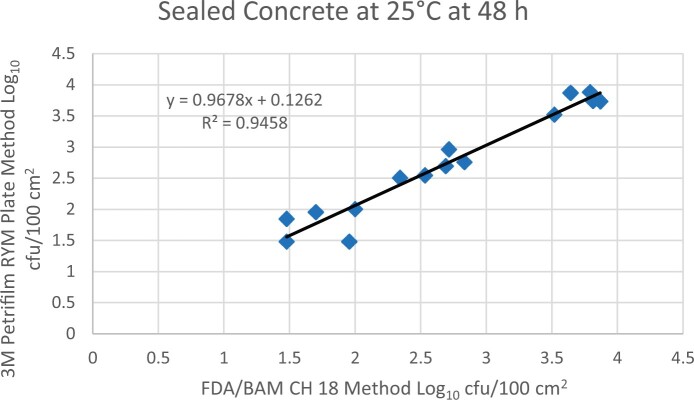
Method comparison results of 3M Petrifilm RYM Plate versus FDA BAM Ch. 18 for sealed concrete at 25°C for 48 h.

**Figure 10.**
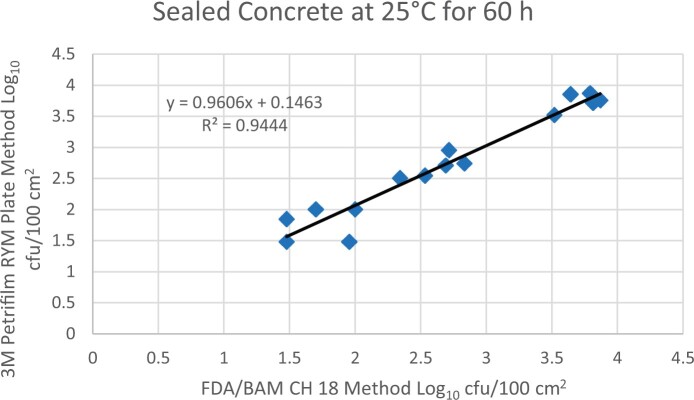
Method comparison results of 3M Petrifilm RYM Plate versus FDA BAM Ch. 18 for sealed concrete at 25°C for 60 h.

**Figure 11.**
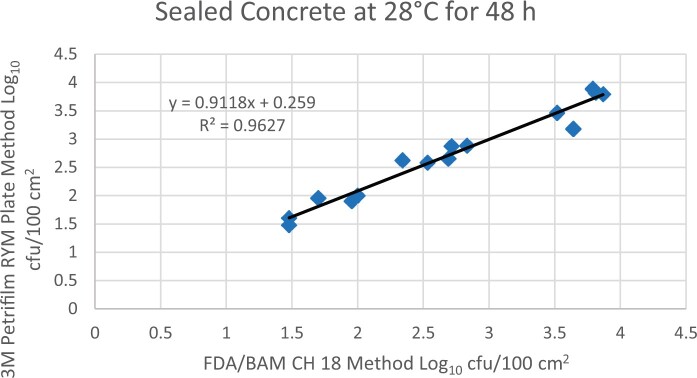
Method comparison results of 3M Petrifilm RYM Plate versus FDA BAM Ch. 18 for sealed concrete at 28°C for 48 h.

**Figure 12.**
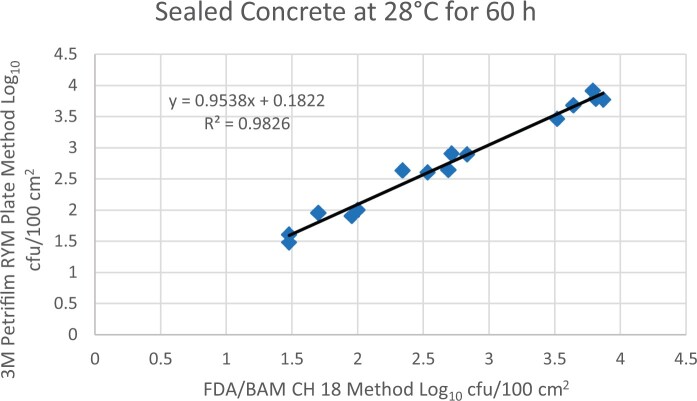
Method comparison results of 3M Petrifilm RYM Plate versus FDA BAM Ch 18 for sealed concrete at 28°C for 60 h.

Prior to inoculation, yeast, and mold results of <10 cfu/test area were obtained for all three environmental surfaces after chemical disinfection. Statistical analysis was conducted for each yeast and mold contamination level for each matrix. Logarithmic transformation of the counts (cfu/test area) was performed and the difference of means, with 90 and 95% confidence intervals (CIs), between the candidate method and the reference method was determined for each matrix and each contamination level. The difference of means and CIs were calculated using the Paired Method Analysis for Micro Testing Version 1.2 (Least Cost Formulations, Ltd, Virginia Beach, VA, USA). The 90 and 95% CI of the bias between the two methods fell between −0.5 to 0.5 log_10_ for each concentration indicating equivalence between the two methods and exceeded the minimum AOAC SMPR recommended acceptance criteria (90% CI of the bias between the two methods is between −0.5 to 0.5 log_10_; 12). The repeatability, calculated as standard deviation, of the 3M Petrifilm RYM Plate and the reference method was determined for all environmental surfaces. Cochran and Grubbs outlier tests were performed for the 3M Petrifilm RYM Plate and the reference method results. The statistical analysis between the 3M Petrifilm RYM Plate and the reference method indicated that no outliers were detected, and that the methods show no statistically significant differences, with 95% confidence. A summary of the study data, statistical analysis and 90 and 95% CIs are presented in Tables 1–4. Figures 1–12 display graphs of the log_10_ values of the candidate method and the reference method.

## Discussion

The 3M Petrifilm RYM Plate method evaluated in this study proved to be a reliable alternative method at both incubation times and temperatures evaluated compared to the BAM Ch. 18 reference method for enumeration of yeast and mold on stainless steel, rubber, and sealed concrete surfaces. The results of the statistical analysis using the difference of means with calculated 90 and 95% CIs indicated equivalence between the 3M Petrifilm RYM Plate method and the reference method in all of the artificial contamination levels analyzed.

The method allows the user to obtain accurate results within 48–60 h in the matrixes evaluated for the presence of yeast and mold when incubated at 25 ± 1°C or 28 ± 1°C. Sample preparation is paired with the reference method, as is the workflow for plating onto the 3M Petrifilm RYM Plate. The 3M Petrifilm Flat Spreader was easy to use and aided the spread of the inoculum across the entire gel surface of the plate. Interpretation and colony counting was straightforward just as with traditional plates. The 3M Petrifilm RYM Plate method required no additional agar or Petri dishes, creating an easier workflow by cutting down on supplies, sample plating time, and most noticeably, occupying less space in the incubator.

## Conclusions

The data from this study support the product claim that the 3M Petrifilm RYM Plate method can be utilized to enumerate yeast and mold from stainless steel, rubber, and sealed concrete environmental surface samples. The method allows the end user to plate samples and transfer them into the incubator faster than the traditional method following BAM Ch. 18, allowing laboratories to process more samples in a shorter period, while still obtaining statistically equivalent results for the matrixes evaluated.

## References

[1] Ryu D. , Wolf-Hall C. (2015) in Compendium of Methods for the Microbial Examination of Foods, 5th Ed., Salfinger Y., Tortorello M.L. (Eds), APHA, Washington, DC, pp 277–286. doi:10.2105/MBEF.0222.

[2] Meechan, P.J., & Potts, J. (Eds) (2020) *Biosafety in Microbiological and Biomedical Laboratories*, 6th Ed. https://www.cdc.gov/labs/pdf/SF__19_308133-A_BMBL6_00-BOOK-WEB-final-3.pdf (accessed December 2021)

[3] ISO 21527-1:2008: Microbiology of food and animal feeding stuffs — Horizontal method for the enumeration of yeasts and moulds — Part 1: Colony count technique in products with water activity greater than 0,95. 2008. Geneva, Switzerland

[4] ISO 21527-2:2008: Microbiology of food and animal feeding stuffs — Horizontal method for the enumeration of yeasts and moulds — Part 2: Colony count technique in products with water activity less than or equal to 0,95. 2008. Geneva, Switzerland

[5] U.S. Food and Drug Administration (2001) *Bacteriological Analytical Manual* (Last updated 10/21/2017) Ch. 18, Yeast Molds and Mycotoxins, https://www.fda.gov/food/laboratory-methods-food/bam-chapter-18-yeasts-molds-and-mycotoxins (accessed October 2021)

[6] *Standard Method Performance Requirements* (SMPR^®^) for Viable Yeast and Mold Count Enumeration in Cannabis and Cannabis Products (AOAC SMPR 2021.009). 2021. https://www.aoac.org/wp-content/uploads/2021/06/SMPR-2021_009.pdf (accessed April 2021)

[7] 3M™ Petrifilm™ Rapid Yeast and Mold Count Plate (RYM) AOAC^®^*Performance Tested Methods*^SM^ Certificate No. 121301. PTM Validated Methods. 2021. https://members.aoac.org/AOAC/PTM_Validated_Methods.aspx (accessed April 2021)

[8] Bird P. , Flannery J., Crowley E., Agin J., Goins D., Jechorek R. (2015) J. AOAC Int. 98, 767–783 26086256 10.5740/jaoacint.15-006

[9] EN ISO 16140 Validation of 3M™ Rapid Yeast and Mold Petrifilm™ plate for yeasts and molds enumeration in food, environmental samples, pet food and animal feed. Certificate: 3M 01/13-07/14. Food industry – NF Validation EN. 2022. https://nf-validation.afnor.org/wp-content/uploads/2014/10/Synt-3M-01-13-07-14_en.pdf (accessed April 2021)

[10] *Official Methods of Analysis* (2019), 21st Ed., AOAC INTERNATIONAL, Rockville, MD, Method **2014.05**, https://www.eoma.aoac.org/gateway/readFile.asp?id=2014_05.pdf (accessed April 2021)

[11] *Official Methods of Analysis* (2019), 21st Ed., Appendix J, AOAC INTERNATIONAL, Rockville, MD, http://www.eoma.aoac.org/app_j.pdf (accessed October 2021)

[12] *Standard Method Performance Requirements* (SMPR^®^) for Quantitative Microbiology Methods for Food and Environmental Samples (Draft document, Version 4, March 11, 2020). 2020. https://www.aoac.org/wp-content/uploads/2020/06/AOAC_Quant_Micro_Methods_SMPRv4_FINAL.pdf (accessed October 2021)

